# Langzeitauswertung der Low-dose-rate(LDR)-Brachytherapie des lokal begrenzten Prostatakarzinoms

**DOI:** 10.1007/s00120-023-02099-0

**Published:** 2023-05-31

**Authors:** N. Carl, J. Rassweiler, J. Andreas, S. Carl

**Affiliations:** 1 Klinik für Urologie und Urochirurgie, Universitätsklinikum Mannheim, Medizinische Fakultät Mannheim, Universität Heidelberg, Theodor-Kutzer-Ufer 1–3, 68167 Mannheim, Deutschland; 2 ehem. Klinik für Urologie und Kinderurologie, SLK-Kliniken Heilbronn, Heilbronn, Deutschland; 3 Urologische Belegabteilung, Kreiskrankenhaus Emmendingen, Emmendingen, Deutschland

**Keywords:** Strahlentherapie, Jod 125, Lokaltherapie, Radioonkologie, Retrospektiv multizentrisch, Radiation therapy, Iodine 125, Local therapy, Radiation oncology, Retrospective Multicenter study

## Abstract

**Hintergrund und Fragestellung:**

Im Zuge von technischen Innovationen, d. h. Verbesserung der Seed-Qualität, der Implantationsmethode und der Bestrahlungsplanung, konnten sich die Behandlungsergebnisse der Brachytherapie stetig verbessern. Randomisierte Kontrollstudien haben gezeigt, dass beim Niedrig-Risiko-Prostatakarzinom kein Unterschied im biochemischen rezidivfreien Überleben (bRFS) zwischen radikaler Prostatektomie und Low-dose-rate(LDR)-Brachytherapie besteht. Ziel dieser Auswertung war die onkologische Wirksamkeit der LDR-Brachytherapie bei Patienten mit lokal begrenzten Prostatakarzinomen.

**Studiendesign und Untersuchungsmethoden:**

Eine retrospektive multizentrische Analyse wurde an 618 Patienten, die zwischen 2004 und 2019 in zwei Zentren in Deutschland mit einer LDR-Brachytherapie-Monotherapie behandelt wurden, durchgeführt. Die Therapie wurde mit Jod^125^-Seeds durchgeführt. Postinterventionell wurden nach 3, 6, 12 Monaten und anschließend jährlich Bestimmungen des prostataspezifischen Antigens (PSA) durchgeführt. Primärer Endpunkt war das bRFS. Die Kaplan-Meier-Methode wurde genutzt und ein biochemisches Rezidiv wurde definiert als der PSA-Anstieg um ≥ 2 ng/ml nach zuvor gemessenem Nadir (Phoenix-Definition).

**Ergebnisse:**

Die mediane Nachbeobachtungszeit betrug 52 (3–180) Monate, das bRFS betrug 87,87 % für die Gesamtkohorte. Besseres bRFS wurde bei Patienten mit Gleason Score (GS) ≤ 7a beobachtet (*p* < 0,0001). Es bestand kein signifikanter Unterschied zwischen GS 6 und 7a. D’Amico-Risikogruppe, initialer PSA-Wert sowie der Anteil karzinompositiver Stanzbiopsien hatten einen signifikanten Einfluss auf bRFS (*p*-Wert < 0,0001; < 0,0001 und 0,0005). Prostatavolumen und ein- oder beidseitiger Befall der Prostata hatten keinen signifikanten Einfluss auf bRFS (*p*-Wert = 0,86 und 0,28).

**Schlussfolgerung:**

Unsere Ergebnisse zeigen eine gute onkologische Wirksamkeit der LDR-Brachytherapie bei Patienten mit einem GS 6 und 7a.

## Abstract (en)

**Background and objectives:**

As a result of technical innovation, i.e., improvement of seed quality, implantation method, and dose calculation, it has been possible to continuously improve oncological results in the treatment of localized prostate cancer with low-dose-rate brachytherapy (LDR-BT). Randomized controlled trials have shown that there is no significant difference in oncological control between the use of radical prostatectomy and LDR-BT in patients with low-risk prostate cancer. The objective of this study was to investigate the oncological efficacy of LDR-BT.

**Materials and methods:**

A retrospective multicenter analysis was conducted on 618 patients treated with LDR-BT as monotherapy, who received a dose of 145 Gy. We used iodine^125^ as the radioactive source. The analysis was conducted with follow-up data from two brachytherapy centers in Germany between 2004 and 2019. The primary endpoint was biochemical relapse-free survival (bRFS), whereby the Phoenix definition (PSA − nadir +2 ng/ml; PSA: prostate-specific antigen) was used to define biochemical relapse, i.e., therapeutic failure.

**Results:**

Median follow-up was 52 months (range 3–180 months). The bRFS across all risk groups was 87.87%. Oncological efficacy was significantly higher in patients with a Gleason score of 6 and 7a (*p*-value < 0.0001); however, there was no significant difference in bRFS between these two groups. Bilateral tumor infiltration or prostate volume had no significant influence on bRFS.

**Conclusion:**

Our results show no difference in bRFS between Gleason score 6 and 7a.

## Hintergrund und Fragestellung

Die Brachytherapie ist eine seit nunmehr Jahrzehnten bekannte Form der Strahlentherapie beim lokal begrenzten Prostatakarzinom. Die Bestrahlung erfolgt über radioaktive Isotope, die in einer kurzzeitigen Operation transperineal in das Prostatagewebe implantiert werden. Der Vorteil dieser Methode resultiert aus dem minimal-invasiven und organerhaltenden Vorgehen, welches lange Krankenausaufenthalte obsolet macht und dabei die perioperative Morbidität und postoperative Nebenwirkungen (z. B. Inkontinenz und erektile Dysfunktion) reduzieren soll. Aktuell existieren zwei Formen der Brachytherapie. Die High-dose-rate(HDR)-Brachytherapie verwendet Isotope mit hoher Radioaktivität wie z. B. Iridium^192^, welche in „Afterloading“-Technik kurzzeitig intraoperativ appliziert werden. Die Low-dose-rate(LDR)-Brachytherapie verwendet Isotope mit einer geringeren Radioaktivität, z. B. Palladium^95^ oder Jod^125^, und die Strahlenquellen verbleiben permanent nach Implantation.

Die AWMF-S3-Leitlinie empfiehlt die Anwendung der LDR(low dose rate)-Brachytherapie als Monotherapie bei lokal begrenzten Prostatakarzinomen der niedrigen Risikogruppe mit einem Gleason Score (GS) von 6 (3 + 3). Die EAU empfiehlt die LDR-Brachytherapie zudem bei GS von 7a (3 + 4). Darüber hinaus empfehlen beide Leitlinien die Brachytherapie in Kombination mit einer externen perkutanen Bestrahlung der Prostata bei Patienten der intermediären und hohen Risikogruppe als alternative Behandlungsmethode [1, 2].

Einige Studien haben die LDR-Brachytherapie bereits retrospektiv und prospektiv untersucht und zeigten gute onkologische Ergebnisse. In Tab. 2 sind die Ergebnisse einiger Studien anhand des biochemischen rezidivfreien Überlebens (bRFS (biochemical relapse free survival) i.e. PSA(Prostata-spezifisches Antigen)-freies Überleben) in einer Übersicht dargestellt.

Die Studie mit dem längsten Beobachtungszeitraum wurde 2011 von der Seattle-Group um Sylvester et al. publiziert. Die prospektiv angelegte Studie untersuchte 215 Patienten der niedrigen, intermediären und hohen Risikogruppe nach D’Amico [3]. Mit einem medianen Nachbeobachtungszeitraum von 15,4 Jahren zeigte sich eine biochemische rezidivfreie Überlebenswahrscheinlichkeit von 85,9 %, 79,9 % und 62,2 % respektive.

Die ersten Langzeitergebnisse zur LDR-Brachytherapie aus dem deutschsprachigen Raum kamen 2020 aus der Schweiz. In einer prospektiven multizentrischen Studie von Viktorin-Baier et al. wurden 1291, mit einer LDR-Brachytherapie behandelten Patienten der niedrigen und intermediären Risikogruppe untersucht. Die mediane Nachbeobachtungszeit lag bei 37,1 Monaten. Nach 7 Jahren zeigte sich bei der niedrigen Risikogruppe ein bRFS von 94 %, sowie 83 % für Patienten mit einem intermediären Risiko. Signifikant höhere biochemische Rezidivraten traten bei GS 7b (4 + 3) auf. Insgesamt erwies sich die LDR-Brachytherapie als effektive Behandlungsoption bei Patienten mit einem GS von 6 sowie 7a [4].

Die genannten beispielhaften Studien untersuchten lediglich die onkologischen Ergebnisse der LDR-Brachytherapie. Vereinzelte Arbeiten, die Behandlungsalternativen mit der LDR-Brachytherapie verglichen haben, sollen hier kurz erläutert werden.

Aktuell existieren 2 abgeschlossene randomisierte Kontrollstudien („randomized control trials“ [RCT]), die die LDR-Brachytherapie (LDR-BT) mit der radikalen Prostatektomie (RP) hinsichtlich der onkologischen Effektivität untersucht haben. Die beiden von Giberti et al. im Jahr 2009 und 2017 publizierten Arbeiten zeigten für das Niedrig-Risiko-Prostatakarzinom keinen signifikanten Unterschied im bRFS zwischen den beiden Behandlungsverfahren mit 91,7–96,1 % (LDR-BT) vs. 91–94,4 % (RP; [5, 6]).

In der Behandlung von Intermediär- und Hoch-Risiko-Patienten zeigte ein 2016 publizierter Zwischenbericht der ASCENDE-RT-Studie signifikante Unterschiede zwischen externer perkutaner Strahlentherapie der Prostata („external beam radiation therapy“ [EBRT]) und der LDR-Brachytherapie. In dieser RCT wurde eine Kohorte von 398 Männern einem „EBRT-Arm“ oder einem „EBRT + LDR-Brachytherapiearm“ zugeführt. Das geschätzte bRFS (nach Kaplan-Meier-Methode) lag bei der Brachytherapiegruppe zwischen 83–89 % vs. 62–84 % für die EBRT-Gruppe. In Abhängigkeit von der Nachbeobachtungszeit zeigte sich ein signifikanter Unterschied im bRFS zugunsten der Kombinationstherapie aus LDR-BT und EBRT. Bemerkenswerterweise kam es nach dem vierten postinterventionellen Jahr zu einer signifikanten Zunahme an biochemischen Rezidiven im EBRT-Arm. Nach einem medianen Nachbeobachtungszeitraum von 6,5 Jahren zeigte die Brachytherapiegruppe verglichen mit der EBRT-Gruppe eine doppelt so hohe Wahrscheinlichkeit der biochemischen Rezidivfreiheit (HR (Hazard Ratio) 2,04; *p* = 0,004; [7]).

Abgeschlossene vergleichende RCT, die die vier etablierten Behandlungsoptionen (AS (Active Surveillance), EBRT, BT und RP) beim lokal begrenzten Prostatakarzinom der niedrigen und intermediären Risikogruppe untersucht haben, existieren aktuell nicht. Die PREFERE-Studie und andere RCT, die sich diese Fragestellung zum Ziel genommen haben, mussten aufgrund von Rekrutierungsproblemen abgebrochen werden [8].

Die Datenlage zur LDR-Therapie v. a. im Vergleich zu anderen Therapieoptionen ist heterogen und die Beurteilung dadurch erschwert (Tab. 1).

**Table 1:** 

Autoren	*n*	Biochemisches rezidivfreies Überleben (%) nach Risikogruppen				Zeitraum (Jahre)	Design
		„Low“	„Intermediate“	„High“	Gesamt		
Potters et al. (2005) [10]	1449	89	78	63	–	12	Retro
Zelefsky et al. (2007) [11]	2693	–	–	–	93	8	Retro
Giberti et al. (2009) [5]	100	–	–	–	91,7	5	RCT
Stone et al. (2010) [12]	584	–	–	–	84,7	7	Retro
Munro et al. (2010) [13]	187	–	82,4	–	–	10	Retro
Sylvester et al. (2011) [3]	3262	–	–	–	80,4	15	Prospektiv
Kittel et al. (2015) [14]	1989	^–^	–	–	81,5	7	Prospektiv
Giberti et al. (2017) [6]	165	–	–	–	97,4	4	RCT
Gestaut et al. (2017) [15]	359	^–^	–	–	89,6	5	Retro
Langley et al. (2018) [16]	597	95	90	87	–	10	Retro
Lazarev et al. (2018) [17]	757	86	80	65	–	10	Retro
Viktorin-Baier et al. (2020) [4]	1291	94	83	–	–	7	Prospektiv
Goy et al. (2021) [18]	110	–	–	–	80,2	10	Retro
Uribe-Lewis et al. (2021) [19]	2936	–	–	–	93^a^	10	Prospektiv
Eigene Ergebnisse (2022)	618	90	90	68,5	87,8	10	Retro

*RCT* „randomized control trial“

^a^*csSP* „cancer-specific survival probability“, krebsspezifische Überlebenswahrscheinlichkeit (Mortalität)

Bei der Anwendung der LDR-Brachtherapie als kurative Behandlungsoption des lokal begrenzten Prostatakarzinoms herrscht eine Diskrepanz in den Empfehlungen zwischen deutscher und europäischer Leitlinie. Die Empfehlungen der deutschen Leitlinie basieren auf eine durch den Gemeinsamen Bundesausschuss (G-BA) beauftragte Risiko-Nutzen-Analyse, die im September 2020 herausgegeben wurde. Bereits dort wurde die Anwendung der LDR-Brachytherapie beim Prostatakarzinom mit einem GS von 7a kontrovers diskutiert [9]. Die von Viktorin-Baier et al. vorgelegte Studie, welche nahezu idente Wirksamkeit bei Patienten mit einem GS von 6 wie 7a aufweist, veranlasste uns diese Ergebnisse an einer deutschen Kohorte zu prüfen.

## Studiendesign und Untersuchungsmethoden

Wir führten eine multizentrische retrospektive Studie an 733 mit einer LDR-Brachytherapie behandelten Patienten mit lokal begrenztem Prostatakarzinom durch. Ein Zeitraum von 2004 bis 2019 wurde festgelegt. Die Eingriffe wurden an der Klinik für Urologie und Kinderurologie der SLK-Kliniken Heilbronn sowie der urologischen Belegabteilung des Kreiskrankenhauses Emmendingen mit identischer Bestrahlungstechnik und Seed-Aktivität durchgeführt. Die erfassten Parameter beinhalteten: Alter, GS, initialer prostataspezifischer Antigenwert (iPSA), positive Prostatastanzbiopsien, Prostataseitenbefall, Prostatavolumen sowie PSA-Verlaufswerte. Serum PSA-Werte wurden 3, 6, 12 Monate nach Implantation und anschließend jährlich für die Auswertung erhoben. Patienten mit mindestens 6 Monaten Nachbeobachtungszeit wurden in die Untersuchung aufgenommen. Die „Nadir +2 ng/ml“-Definition (Phoenix-Definition) wurde als Definition für ein biochemisches Rezidiv festgelegt. Als primärer Endpunkt wurde das bRFS ausgewertet. Die Kohorte wurde anhand der in Tab. 2 aufgelisteten Untersuchungsparameter stratifiziert und individuelle Faktoren mit Einfluss auf das bRFS untersucht. Die bRFS der Kohorte und der Subgruppen wurde mit der Kaplan-Meier-Methode für die Parameter mit signifikantem Einfluss (*p*-Wert < 0,05) berechnet. Außerdem wurden multivariate und univariate Regressionsanalysen sowie die Berechnung von Hazard Ratios (HR) nach Cox-Regressionsmodell durchgeführt.

**Table 2:** 

				Univariate Cox-Regression				Multiple Cox-Regression			
Variabel	Level	*n*	Biochemisch rezidiv	HR	95 %	KI	*p*	HR	95 %	KI	*p*
Alter bei Therapiebeginn (Jahre)	< 65	164	19	Ref	–	–	*0,03*	–	–	–	**–**
	65–69	149	19	1,18	0,62	2,22	–	–	–	–	–
	70–74	162	14	0,79	0,40	1,57	–	–	–	–	–
	75 +	143	23	*2,02*	1,09	3,71	–	**–**	–	–	–
Prostatavolumen (ml)	< 27	130	18	1,03	0,55	1,95	0,86	–	–	–	–
	27–34,9	150	21	1,10	0,59	2,02	–	–	–	–	–
	35–43,9	131	15	0,82	0,42	1,61	–	–	–	–	–
	44 +	156	20	Ref	–	–	–	–	–	–	–
Initiales PSA (ng/ml)	< 10	460	45	Ref	–	–	*<* *0,0001*	–	–	–	–
	10–20	102	18	*2,76*	1,59	4,79	–	–	–	–	–
	20 +	48	12	*3,72*	1,96	7,05	–	–	–	–	–
Gleason Score	< 6	37	2	0,39	0,09	1,67	*<* *0,0001*	0,39	0,09	1,67	*<* *0,0001*
	6	211	22	1,08	0,59	1,96	–	1,08	0,59	1,96	–
	7a	269	21	Ref	–	–	–	Ref	–	–	–
	7b	66	13	*2,70*	1,35	5,40	–	*2,70*	1,35	5,40	–
	8	9	3	*5,80*	1,72	19,5	–	*5,80*	1,72	19,5	–
	9–10	25	14	*12,5*	6,28	24,7	–	*12,5*	6,28	24,7	–
Gleason Group nach: ISUP 2014/ WHO 2016	1	248	24	0,94	0,52	1,69	*<* *0,0001*	–	–	–	–
	2	269	21	Ref	–	–	–	–	–	–	–
	3	66	13	*2,70*	1,35	5,39	–	–	–	–	–
	4	9	3	*5,77*	1,71	19,4	–	–	–	–	–
	5	25	14	*12,4*	6,25	24,6	–	–	–	–	–
D’Amico Group	„Low“	211	21	0,75	0,43	1,30	**–**	–	–	–	–
	„Intermediate“	331	32	Ref	–	–	–	–	–	–	–
	„High“	70	22	*4,17*	2,42	7,18	*<* *0,0001*	–	–	–	–
Anteil positive Biopsien (%)	< 10	77	8	1,56	0,65	3,71	*0,0005*	–	–	–	–
	10–19	123	11	1,00	0,46	2,18	–	–	–	–	–
	20–39	156	15	Ref	–	–	–	–	–	–	–
	>40	136	28	*3,19*	1,69	6,03	–	–	–	–	–
Befall der Seitenlappen	Einseitig	296	35	Ref	–	–	0,28	–	–	–	–
	Beidseitig	195	27	1,32	0,80	2,18	**–**	–	–	–	–

*HR* Hazard Ratio, *ISUP* International Society of Urological Pathology, *KI* Konfidenzintervall, *PSA* prostataspezifisches Antigen

### Bestrahlungstechnik

Als Strahlenquelle diente das radioaktive Isotop Jod^125^. Das in Seeds enthaltene Jod^125^ wurde mit der „4D-Methode“ über Hohlnadeln transperineal in die Prostata implantiert.

**Figure 1:**
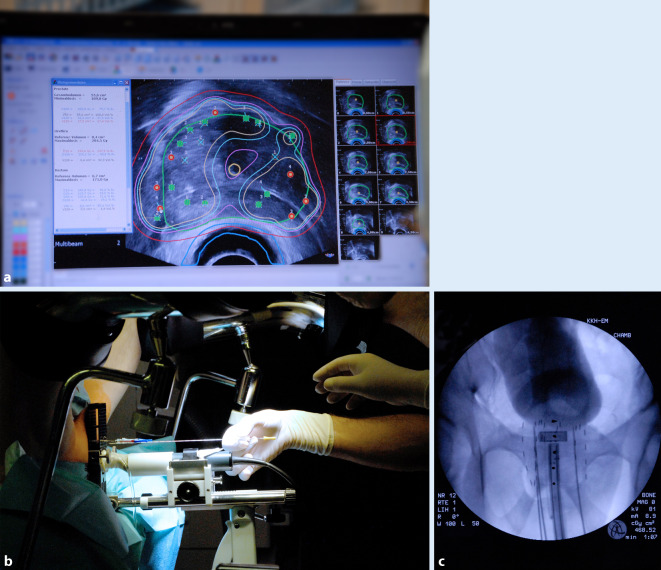

Die 4D-Methode ergibt sich aus der 3D-Planung des Zielvolumens (i.e. Prostata) und der Ablage der Seeds unter „Echtzeitbedingung“. Zunächst erfolgte die 3D-Planung mit dem transrektalen Ultraschall (TRUS) der Prostata zur Festlegung des Zielvolumens, anschließend die Template- und TRUS-gesteuerte Implantation. Mit der 4D-Methode konnte vor Ablage der Seeds die Lokalisation jeder Nadel durch das TRUS-Bild im Planungssystem lokalisiert werden und die geplante mit der tatsächlichen Position in Deckung gebracht werden. Durch die Darstellung der „Ist-Soll-Positionen“ in der Planungssoftware (Abb. 1) wurden die Isodosis der Prostata und Referenzdosen der Risikoorgane (Harnröhre und Rektum) analog zur Implantation der Seeds laufend neu berechnet und überprüft. Bei Unterschreitung der Zieldosis konnten die verbleibenden Nadelpositionen gegeben Falls angepasst werden, um die Isodosis von 145 Gy (Gray) unter Schonung der umliegenden Risikoorgane (Harnröhre und Rektum) zu erreichen. In der Regel betrug die Operationszeit 45–60 min.

## Ergebnisse

Zwischen September 2004 und September 2019 konnte von insgesamt 733 behandelten Patienten 618 mit ≥ 2 postoperativen PSA-Bestimmungen ausgewertet werden. Die Patientencharakteristika der untersuchten Kohorte sind in Tab. 2 dargestellt. Der mediane Nachbeobachtungszeitraum lag bei 52 (6–180) Monaten.

Der GS hatte erwartungsgemäß einen hochsignifikanten Einfluss auf das Auftreten eines biochemischen Rezidivs (*p* < 0,0001). Die multivariate Analyse zeigte, dass der GS den höchsten statistischen Einfluss auf das onkologische Ergebnis hatte.

Der Großteil der Kohorte (83,6 %) erschloss sich aus Patienten der niedrigen und intermediären Gruppe mit einem GS von 6 (40,1 %) und 7a (43,5 %). Über den gesamten Beobachtungszeitraum trat in absoluter Zahl bei 9,68 und 7,81 % respektive ein biochemisches Rezidiv nach Phoenix-Definition auf. Bei Patienten mit einem GS von 7b konnte eine deutliche Zunahme der Rezidivraten beobachtet werden. Von *n* = 66 Patienten zeigte sich bei 19,7 % ein biochemisches Rezidiv, im Vergleich zu Referenzgruppe war das Risiko hier um 170 % erhöht (HR =2,7; *p* < 0,0001; KI = 1,35:5,4).

**Figure 2:**
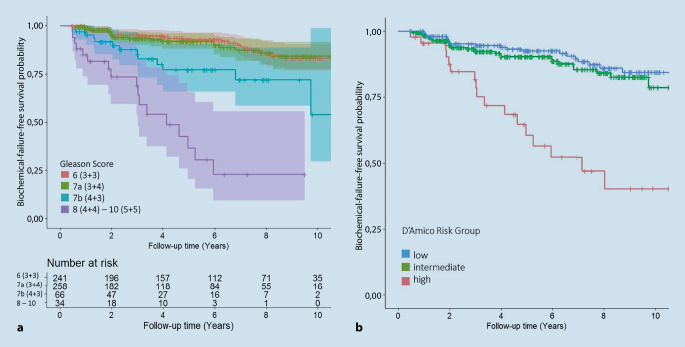

Die Analyse der Gesamtkohorte zeigte, dass die Kaplan-Meier-Kurven für GS 6 und 7a über den dargestellten Zeitraum annährend gleich verliefen (Abb. 2). Die biochemische rezidivfreie Überlebenswahrscheinlichkeit für Patienten mit GS von 6 und 7a lag bei > 90 % und es bestand kein signifikanter Unterschied zwischen beiden Gruppen.

In der Auswertung der Gesamtkohorte der Risikogruppen nach D’Amico konnte bei Patienten der niedrigen Risikogruppe in 9,95 % und bei Patienten der intermediären Risikogruppe in 9,67 % der Fälle ein biochemisches Rezidiv beobachtet werden. Die Risikogruppierung hatte einen signifikanten Einfluss auf das Auftreten eines bioschemischen Rezidivs (*p* < 0,0001), wobei sich zwischen der niedrigen und intermediären Risikogruppe kein signifikanter Unterschied im bRFS zeigte.

Die Seitenausdehnung des Prostatakarzinoms innerhalb der Prostata hatte laut unserer Auswertung keinen signifikanten Einfluss auf das bRFS (*p*-Wert =0,28). Beim Prostatavolumen konnte ebenfalls kein signifikanter Unterschied beobachtet werden. Auch bei großen Prostatadrüsen von bis zu 100 ml zeigte sich kein signifikanter Anstieg an biochemischen Rezidiven (*p*-Wert =0,86).

## Diskussion

Trotz kontinuierlicher Verbesserung des technischen Verfahrens und guten Langzeitresultaten kommt die LDR-Brachytherapie, verglichen mit der radikalen Prostatektomie und der externen Strahlentherapie, bei der Behandlung des Prostatakarzinoms in Deutschland nur in begrenztem Umfang zur Anwendung.

In den USA herrschte zuvor ein umgekehrtes Verhältnis von Strahlen- und operativer Therapie. Im Zeitraum von 2004 bis 2011 wurden in den USA etwa zwei Drittel aller Patienten mit einer primären Strahlentherapie behandelt, wohingegen ein Drittel eine RP erhalten haben. Im selben Zeitraum erhielten in Deutschland 10 % eine primäre Strahlentherapie und 66 % eine RP [20]. Aktuellere Daten aus den USA zeigen die Abnahme von zuvor 25 auf 16 % mit einer LDR-Brachytherapie behandelten Patienten [21]. Gründe für diesen Trend können die zunehmende Beliebtheit der roboterassistierten laparoskopischen Prostatektomie sowie die in internationalen Leitlinien fest verankerte aktive Überwachung sein. Zuletzt wurde bei der aktiven Überwachung das Indikationsspektrum mit der Leitlinie der EAU (2022) auf Patienten mit einem GS von 7a erweitert, was die Konkurrenz bei der Indikationsstellung erhöht [22].

## Fazit für die Praxis

- Die LDR-Brachytherapie zeigt jedoch gute onkologische Ergebnisse bei Patienten mit einem GS von 6 und 7a.
- Das Verfahren der Low-dose-rate(LDR)-Brachytherapie stößt als Monotherapie bei Patienten ab einem Gleason-Score (GS) von 7b an seine Effektivitätsgrenze.
- Unsere Ergebnisse decken sich mit den Ergebnissen der bestehenden Literatur und zeigen, dass die LDR-Brachytherapie beim richtigen Indikationsspektrum eine sichere Behandlungsalternative sein kann.
